# Ten Simple Rules for Organizing a Virtual Conference—Anywhere

**DOI:** 10.1371/journal.pcbi.1000650

**Published:** 2010-02-26

**Authors:** Nelson N. Gichora, Segun A. Fatumo, Mtakai V. Ngara, Noura Chelbat, Kavisha Ramdayal, Kenneth B. Opap, Geoffrey H. Siwo, Marion O. Adebiyi, Amina El Gonnouni, Denis Zofou, Amal A. M. Maurady, Ezekiel F. Adebiyi, Etienne P. de Villiers, Daniel K. Masiga, Jeffrey W. Bizzaro, Prashanth Suravajhala, Sheila C. Ommeh, Winston Hide

**Affiliations:** 1 International Institute of Tropical Agriculture, Nairobi, Kenya; 2 Faculty of Life Sciences, The University of Manchester, Manchester, United Kingdom; 3 Department of Computer and Information Sciences, Covenant University, Ota, Nigeria; 4 Institute of Bioinformatics, Johannes Kepler University, Linz, Austria; 5 Moroccan Society for Bioinformatics Institute, Morocco; 6 South African National Bioinformatics Institute, University of the Western Cape, Bellville, South Africa; 7 University of Cape Town, Cape Town, South Africa; 8 University of Notre Dame, South Bend, Indiana, United States of America; 9 Biotechnology Unit, University of Buea, Buea, South West Region, Cameroon; 10 International Livestock Research Institute, Nairobi, Kenya; 11 Biosciences Eastern and Central Africa, Nairobi, Kenya; 12 International Center of Insect Physiology and Ecology, Nairobi, Kenya; 13 Bioinformatics Organization, Hudson, Massachusetts, United States of America; 14 Bioinformatics Team, Center for Development of Advanced Computing, Pune University Campus, Pune, India; 15 Harvard School of Public Health, Boston, Massachusetts, United States of America; University of California San Diego, United States of America

The *First African Virtual Conference on Bioinformatics 2009* (AFBIX09) [1], organized by the Bioinformatics Organization [2] and the International Society for Computational Biology Student Council's Regional Student Groups of Africa and Morocco (ISCBSC RSG-Africa and RSG-Morocco) [3] received support from the African Society for Bioinformatics and Computational Biology (ASBCB) [4]. The aim was to provide students and scientists in the bioinformatics and computational biology fields a chance to network through a unique platform conceptualized as “hubs.” These hubs then gave participants the opportunity to foster both physical and virtual interactions as well as develop collaborations, irrespective of geographical location.

Virtual conferencing may prove to be an effective low-cost strategy for conveying bioinformatics and computational biology education to African scientists who otherwise would be deprived of the opportunity. Unlike conventional conferences, virtual conferencing permits the involvement of a greater number of participants who would otherwise be unable to participate in events of this breadth owing to (1) limited travel fellowships, if any; (2) lack of time to travel to distant conference locations; and (3) insufficient accommodation and subsistence funds. These factors apply in general to the post-/undergraduate student community and especially to the target audiences that reside in developing countries. Minimizing the requirement to travel also means that the availability of invited speakers is greatly increased, improving the chances of attracting highly relevant and high-impact presenters.

Through the use of video conferencing software, virtual conferences are able to provide an accessible and cost-effective alternative to real time conferences while retaining the key benefits presented by an on-site conference, such as learning opportunities, sharing of ideas, and networking. The use of inexpensive “commodity off-the-shelf” (COTS) technologies permit anyone with an Internet connection, Web cam, and headset to give and/or attend a presentation. According to Andrew Sage, Cisco Systems' vice president for marketing, virtual conferences “can live on long after the physical booths have been torn down,” while content continues to be viewed in a dedicated virtual environment by many people, even after the conclusion of the event [5].

At the Fall Joint Computer Conference on December 9, 1968, Douglas Engelbart presented, among other innovations, a virtual conferencing system that utilized the broadcast of computer monitor video as well as presenter audio and video [6]. This “expensive approach” has involved traditional video conferencing and technologies such as the Access Grid [7], which have been viable options for the most affluent regions of the world, but the approaches mentioned here are broad enough to be used in both developed and undeveloped environments.

The conference was set up as a series of virtual hubs defined as a group of ten or more persons in one location. Each hub consisted of a computer attached to a Web cam and speakers with a stable Internet connection. The hub activities and the interaction with other hubs were coordinated by persons within the locality.

Speakers within faculty and industry were identified on the basis of their expertise or involvement and relevance to the research topics covered by the virtual conference. There were a total of 16 speakers and out of these, four were keynotes divided between 2 days and four sessions. In addition, there were five invited speakers and three oral presentations selected from 12 submitted abstracts. The rest of the abstracts were presented as posters during break sessions. There were tutorials, relevant discussions from senior faculties, as well as welcome and closing statements from AFBIX09 organizers.

The conference was 19 hours long and was held over 2 days. The first day consisted of 8 hours, tailored to accommodate time zone differences between each of the participating hubs. This was inclusive of 100 minutes of break time divided between two 20-minute coffee sessions concurrently spent on poster presentations, with an hour on a lunch break and 20-minute welcome speech. The second day consisted of an 11-hour program including one 20-minute coffee and poster session, 40-minute lunch break, and 30-minute vote of thanks and closing remarks.

The following ten simple rules are derived from experiences gained while organizing AFBIX09. We propose these as reference material to those intending to plan for similar events, with particular emphasis on resource-constrained communities.

## Rule 1: Address time zone differences: timing is everything

Allow between 6 to 9 months before the conference to permit (1) administrators in the respective virtual hubs a sufficient amount of time to finalize their decisions regarding presentation and/or attendance time slots (relative to time zones) and (2) IT departments' confirmation for the provision of necessary support, amongst other logistics, for the designated event times. The organizing committee should agree on a conference schedule that will be suitable for the time zones of all participating groups.

It is effective to create a proposed conference program for all participating groups in their local time zones to avoid confusion. Once established, it is then crucial to conduct tests of the proposed times precisely as scheduled, weeks before the actual event, to ensure the reliability of the conference program and to identify problems that could arise.

## Rule 2: Test the available resources: to ensure that you are able to host the conference

Ensure the availability of (1) a stable Internet connection; (2) a computer installed with the required video-conferencing software; (3) reliable audio speakers that have been tested for audio clarity; (4) adequate screen resolution for the capabilities of the network; and (5) a public-address system (i.e., video camera and projector connections). There should be adequate lighting for the conference hall to avoid glare or other aspects of poor visibility. Another useful resource is a standby computer assigned to the hub-coordinator with a communication application/device, such as a VoIP service, in place to ensure synchronous coordination of the proceedings with other participating hubs.

As an illustration, the last point was particularly useful in an instance where two of the participating hubs during the conference experienced network downtime, cutting off real-time presentations. Before the restoration of network connection, the respective hub coordinators had to inform the other hubs of their downtime and continually synchronize conference activities.

## Rule 3: Manage bandwidth usage: to safeguard against conference interruptions

It is critical and advisable to make sure your organizations' IT personnel are able to allocate sufficient bandwidth to the virtual conference, to avoid disruptions of live presentations (especially in organizations where network resources are shared). Alternatively, if a group of 10 or more participants are registered for the conference, it is advisable that these individuals form an independent virtual hub to save on bandwidth usage. This approach will reduce the number of Internet connections being used and thus the potential complications for your virtual conference while allowing other users an equally reliable functioning network.

## Rule 4: The concept of virtual hubs: makes registration and participation simpler

Distribute the virtual conference registration fee across all participating hubs and participants [8]–[12]. Cumulative hub payments ensure a reduced registration fee for the individual participant. Hubs provide local expertise and relevant local advertising for the conference. These “front porch” gathering sites compensate for some of the personal interaction that can be missing from virtual conferences. The use of virtual hubs as “conference nodes” tends to increase impact by providing access for those without the equipment and also traditional face-to face interaction. Hub participants can also share traditional meeting activities such as enjoying a meal together.

## Rule 5: Prerecord presentations: to gear-up if streaming video fails for any reason

There is a wide range of software available to get connected virtually (e.g., WebEx, Netviewer, Adobe Connect, etc.), however all available Internet systems are subject to bandwidth limitations and resulting congestion. It is therefore advisable that presentations be prerecorded and in no less than 2 weeks before the conference, in order to permit time for the recordings to be edited or redone, if necessary. Prerecorded presentations can then be hosted via the conference Web sites, making them available to the participating groups in an agreeable video format and in good time to conduct/resolve software compatibility concerns. Moreover, this allows the participants a chance to become familiar with the conference content and to play back presentations containing key concepts/information. The use of prerecorded presentations compensates for slow and unreliable networks and even intermittent electrical outages (e.g., when two of the aforementioned hubs experienced connectivity problems, they resorted to projecting prerecorded presentations to the participants in their respective hubs, and when this was resolved they were able to join the live Q&A sessions). Alternatively, if the network problems are not restored in time, the narrator can then appear online after the prerecorded presentation to answer questions in real time or to take questions via a text-based chat system.

## Rule 6: Allocate time for presenter orientation: to ensure glitch-free schedule compliance

Keynote and invited presenters should become familiar with the designated software, preferably a month before the conference. This will enable them to get acquainted with the software while allowing them to prerecord their own presentation at their convenience. Recorded presentations should then be sent to the conference host, who should test and archive all recordings before use if/when the scheduled presenter is absent at the time of his/her presentation.

## Rule 7: Establish dedicated virtual interaction rooms (e-lobbies): to ensure a practical platform for participant Q&A and networking

Each participating hub should have at least one person responsible for the collection and consolidation of all participant questions or answers from that hub. This consolidation avoids redundancy while saving time and kilobytes. Alternatively, the designated person could verbally relay the questions to the presenters on behalf of the hub to ensure clarity. This approach is especially applicable in cases where one of the hubs is in a country where the language of instruction is not the one adopted for the conference. The availability of “e-lobbies” will permit the comfortable virtual interaction of participants with similar research interests during virtual poster sessions and/or coffee breaks.

## Rule 8: Troubleshoot technical glitches: to equip yourself for any foreseeable challenges

Identify at least one person per hub to coordinate the technical set-up of the conference venue and to ensure, well in advance, that all technical equipment and relevant software are available and functioning properly.

## Rule 9: Get motivated… It's the key to your success

It is crucial to be able to set and meet your deadlines/milestones through adequate time management, hub organization, etc. Besides this, involve people who are inspired, willing, and passionate to organize the conference. Encourage participants in different hubs to take photos throughout the event. The effects of team building last long after the conference, and encouraging participation results in leadership development. Plus, the managerial skills developed play an enormous part in the success of the conference.

## Rule 10: Participant feedback: useful for future reference

At the conclusion of the conference, be sure to request feedback from the participants to be able to identify any faults or errors that can then be addressed in future events. Make sure to have all questions that were raised during the presentations and their corresponding answers available online to all participants including photos taken during the event. Aside from having this information on record, it will help sustain communication even after the virtual conference has been concluded.

The recorded videos and presentations have been made available through Bioinformatics.Org and hyperlinked on the wiki page at http://www.bioinformatics.org/wiki/Afbix09. Bioinformatics.Org seeks the opinions of the community via online polls. Blogging was not implemented in this conference, but we envisage that the online educational system operated at Bioinformatics.Org could be utilized for that in the future.

## Valuable Lessons

Overall, what worked included prerecording the presentations, which were of great assistance when streaming video failed. Use of a chat facility (e.g., Skype) was key in coordinating hub activities during the course of the conference as some of the participating hubs experienced connectivity problems and had to synchronize their prerecorded presentation with the live presentations being viewed by other hubs.

What didn't work included disruption in the streaming video, which was a major drawback, and resulted in most hub coordinators relying on prerecorded videos of the conference presentations. Virtual interaction rooms (e-lobbies) were not effectively utilized as earlier anticipated; this was in contrast to the hub level where participants were able to effectively interact. It would be useful to set up subcommittees in order to deal with conference requirements as they arise. These include technical committees, fundraising committees, and scientific committees among others. It is also important for all committee members to meet regularly with the frequency of meetings increasing as the conference start date draws near.

## Impact on Science in Africa

The novel idea of virtual hubs through e-conferencing was pioneered in AFBIX09. With a stable Internet connection, the maximum number of participants at any conference is dependent on whether future conferences will adopt the concept of virtual hubs. This means that the audio-visual facilities in each hub and sitting space should dictate the maximum number of persons in one hub as compared to the single user participation option. Depending on the choice of the video-conferencing software and the maximum number of connections it can allow at a given time, this value can be translated to hubs. Therefore the number of participants that can attend a virtual meeting will depend on the number of formed hubs and consequently, the maximum capacity of each hub, which may translate to thousands of participants. A new high bandwidth optical fiber cable is being laid around the coast of Africa with bandwidth improvements of 10–100 times expected around most places in Africa. This development should greatly affect future virtual activities within the continent. The African Virtual Conference on Bioinformatics (AFBIX), which was a hybrid between a normal and virtual conference, has had a large impact in the field and consequently there are plans to hold it biennially. This has impacted greatly on ISCB Regional students groups (see below) as well as other spin-off conferences such as the Indian Virtual Conference on Bioinformatics (Inbix10, http://www.bioinformatics.org/wiki/Inbix10).

In terms of participants, the Regional Student Group (RSG)-Moroccan hub had a total of 12 attendees for the AFBIX09, which enabled RSG-Morocco to develop a working relationship/collaboration with the Institut Pasteur de Tunis in Tunisia. The presentations made during the conference sparked discussions between students and scientists touching on the various topics covered, leading to the forging of new ideas on possible bioinformatics projects to undertake.

The RSG-Africa-Southern Africa hub attracted on average ten attendees for the 2 days. The hub was faced with technical issues that affected the quality of the presentations. Although overall, the attendees benefited greatly and called for improvement of future conferences.

The RSG-Africa-Eastern Africa hub attracted a total of 25 attendees as a result of a collaborative effort between the Biosciences East and Central Africa (BecA), who funded all of the students, and the International Livestock Research Institute (ILRI), who provided conferencing facilities gratis. The success of AFBIX09 prompted members to come up with plans to start collaborative bioinformatics projects between RSG-Africa-Eastern Africa and other RSGs, organizations, or institutes that will enable greater collaborations in research and training. The hub also established contacts with RSG-India, which has experience in virtual collaborative bioinformatics projects.

The RSG-Africa-Western Africa hub had a total of 17 attendees. The conference provided a platform for forging collaboration between the biological sciences and computer science departments at Covenant University, which acted as the hub for the conference. The conference attracted key administrators in their institute, including the vice chancellor, and this was a great boost for the students' group of West Africa.

The University of Notre Dame had an average range of eight to 20 attendees.In addition, three other faculties participated in the conference. This was a sure venue to foster collaboration with other students in developing countries.

The total number of participants, including speakers, organizers, and single user participants was close to 100. In conclusion, although several challenges were experienced, AFBIX09 has established a foundation for future virtual conferences.
